# Evolution of New Genotype of West Nile Virus in North America

**DOI:** 10.3201/eid1705.101707

**Published:** 2011-05

**Authors:** Allison R. McMullen, Fiona J. May, Li Li, Hilda Guzman, Rudy Bueno, James A. Dennett, Robert B. Tesh, Alan D.T. Barrett

**Affiliations:** Author affiliations: University of Texas Medical Branch, Galveston, Texas, USA (A.R. McMullen, F.J. May, L. Li, H. Guzman, R.B. Tesh, A.D.T. Barrett);; Harris County Public Health and Environmental Services, Houston, Texas, USA (R. Bueno, Jr., J.A. Dennett)

**Keywords:** West Nile virus, viruses, North America, evolution, genotype, genomic sequences, research

## Abstract

Previous studies of North American isolates of West Nile virus (WNV) during 1999–2005 suggested that the virus had reached genetic homeostasis in North America. However, genomic sequencing of WNV isolates from Harris County, Texas, during 2002–2009 suggests that this is not the case. Three new genetic groups have been identified in Texas since 2005. Spread of the southwestern US genotype (SW/WN03) from the Arizona/Colorado/northern Mexico region to California, Illinois, New Mexico, New York, North Dakota, and the Texas Gulf Coast demonstrates continued evolution of WNV. Thus, WNV continues to evolve in North America, as demonstrated by selection of this new genotype. Continued surveillance of the virus is essential as it continues to evolve in the New World.

West Nile virus (WNV) is a mosquito-borne flavivirus belonging to the Japanese encephalitis serogroup and maintained in an enzootic cycle between mosquitoes (primarily *Culex* spp.) and birds. Mammals such as horses and humans act as dead-end hosts. Most human infections are asymptomatic; West Nile fever develops in ≈20% of infected patients and neuroinvasive disease develops in <1% (*1*).

WNV was first isolated in Uganda in 1937 and was generally associated with sporadic outbreaks of mild, febrile illness until the 1990s, when several epidemics of neuroinvasive disease were reported in northern Africa, eastern Europe, and Russia (*2**–**4*). In 1999, WNV was first isolated in North America from human and bird samples during an outbreak of encephalitic disease in New York. After this outbreak, WNV rapidly spread across the United States north to Canada and south to the Caribbean region, Mexico, and Central and South America.

By 2002, the original WNV genotype isolated in New York, known as NY99, was displaced by a new genotype, designated the North American (NA) or WN02 genotype (hereafter termed NA/WN02 genotype) (*5**,**6*). This genotype is characterized by 13 conserved nt changes, 1 of which results in an amino acid substitution, V159A, in the envelope (E) protein. The NA/WN02 genotype is believed to have become dominant in North America because of its ability to more efficiently disseminate in mosquitoes than the original NY99 virus genotype (*6**–**8*).

Beasley et al. (*9*) first identified the NA/WN02 genotype in Texas in 2002, and further studies showed that this genotype had spread throughout the Upper Texas Gulf Coast and to other regions in the United States (*5*). Additional studies examined phenotypic changes in WNV isolates from the Upper Texas Gulf Coast region during 2003 and identified co-circulation of small-plaque, temperature-sensitive, mouse-attenuated and large-plaque, non–temperature-sensitive, mouse-virulent strains (*10**–**12*). Subsequent studies of the E gene of viruses isolated through 2006 suggested that since the emergence of the NA/WN02 genotype, WNV in North America is either genetically homeostatic (*13*) or its growth rate is decreasing (*14*).

We examined genetic variation in selected WNV strains since 2005 from the Upper Texas Gulf Coast region, in particular, Harris County, Texas, USA (Houston metropolitan area). We report the isolation of genetic variants that demonstrate the continuing evolution of WNV in North America. We also show that the southwestern US genotype first identified in Arizona, Colorado, and northern Mexico in 2003 (termed SW/WN03 genotype) has now spread to the Upper Texas Gulf Coast region.

## Materials and Methods

### Virus Isolates

Virus isolates were obtained from the World Reference Center for Emerging Viruses and Arboviruses at the University of Texas Medical Branch (UTMB) in Galveston, Texas. All new isolates used in this study were originally made from mosquito pools or the brains of naturally infected birds cultured in Vero cells at UTMB. Each isolate was given a second passage in Vero cells to generate a working stock and stored at –80°C.

### Reverse Transcription–PCR

Viral RNA was extracted from 140 μL of infected Vero cell supernatant by using the QIAamp Viral RNA Mini Kit (QIAGEN, Valencia, CA, USA) per the manufacturer’s directions. Full-genome sequencing was performed by consensus overlapping sequencing of PCR products with primers based on the published sequence of WNV NY-99 flamingo 382–99 (GenBank accession no. AF196835). Reverse transcription–PCR was performed by using the Titan One Tube RT-PCR Kit (Roche Applied Science, Indianapolis, IN, USA) (primers and PCR conditions are available by request). PCR products were subjected to electrophoresis on 1% agarose gels and purified by using the QIAquick Gel Extraction Kit (QIAGEN).

### Sequencing and Analysis

Purified PCR products were sequenced in both directions by using the Protein Chemistry or Molecular Genomics Core Laboratories at UTMB. Sequences were edited and assembled by using ContigExpress in the VectorNTI program suite (Invitrogen, Carlsbad, CA, USA). Full-length coding sequences were aligned with all published full-length North American WNV isolate sequences available in GenBank (as of November 2010) and isolate WNV IS-98 STD by using MUSCLE in Seaview version 4 (*15*). The final open reading frame (ORF) alignment contained 244 sequences of 10,299 nt (3,433 aa residues). A second alignment was made by using MUSCLE; this aligment contained 33 sequences from the Upper Texas Gulf Coast region. This alignment contained 11,030 nt and contained the entire ORF and portions of the 3′ and 5′ untranslated region (UTR).

Phylogenetic trees were inferred using the neighbor-joining (NJ) method in the Phylip package (*16*) and the maximum-likelihood (ML) method by using PhyML (*17*). MODELTEST, in conjunction with PAUP, was used to identify generalized time reversible + I + Γ_4_ as the best-fit nucleotide substitution model to be used in phylogenetic analyses (*18**,**19*). To assess robustness of the phylogenetic methods used, we used the NJ method and 1,000 bootstrap replicates. The ML method used 100 bootstrap replicates for the entire North American alignment and 1,000 bootstrap replicates for the Upper Texas Gulf Coast region alignment. IS-98 STD was used as outgroup for the entire North American WNV alignment, and NY99 was used as outgroup for the Upper Texas Gulf Coast region alignment.

### Recombination Detection and Selection Analysis

Screening for recombination was performed on the first 9,999 nt of the North American WNV ORF alignment by using single-break point analysis on the Datamonkey server (*20**–**22*). This screening verified absence of recombination in sequences before running the selection analyses. The first 9,999 nt were selected because of constraints on sequence length by the programs used.

Using the ratio of nonsynonymous (d_N_) to synonymous (d_S_) nucleotide substitutions, we examined the genome for sites of positive selection. Positive selection was defined as d_N_>d_S_ and a p value <1.0. Using the Datamonkey web server (*21**,**22*), we used 3 methods to detect site specific nonneutral selection: single-likelihood counting (SLAC), fixed effects likelihood (FEL), and internal FEL (IFEL) (*23**,**24*). BioEdit was used to create datasets for the first 9,999 nt of the ORF and for each gene (capsid [C], premembrane [prM], E, nonstructural protein 1 [NS1], NS2A, NS2B, NS3, NS4A, NS4B, and NS5) for these analyses (*25*).

## Results

### Viral Isolates

Viruses used in this study were isolated during 2005–2009 from mosquitoes or dead birds collected in Harris County. There were 111 isolates: 14 from 2005, 11 from 2006, 36 from 2007, one from 2008, and 49 from 2009. The genomic sequences of 17 geotemporally representative isolates were determined and compared with other WNV strains from the Upper Texas Gulf Coast region (Harris, Jefferson, and Montgomery Counties) isolated during 2002–2005 and sequenced in our laboratory (Table 1) (*5**,**10**,**12*).

**Table 1:** West Nile viruses from the Upper Texas Gulf Coast, USA, used to study genotype evolution, 2002–2009

Strain	Source	County	Collection year	GenBank accession no.
TX2002–1	Human	Unknown	2002	DQ164198
TX2002–2	Human	Unknown	2002	DQ164205
TVP8533	Human	Jefferson	2002	AY218294
Bird 114	Blue jay	Harris	2002	GU827998
Bird1153	Mourning dove	Harris	2003	AY712945
Bird1171	Great-tailed grackle	Harris	2003	AY712946
v4095	*Culex quinquefasciatus* mosquito	Harris	2003	GU828002
v4380	*Cx*. *quinquefasciatus* mosquito	Harris	2003	GU828001
Bird1881	Mourning dove	Jefferson	2003	GU828003
Bird1519	Blue jay	Montgomery	2003	GU828004
Bird1576	Blue jay	Montgomery	2003	GU827999
Bird1175	Blue jay	Harris	2003	GU828000
Bird1461	Blue jay	Harris	2003	AY712947
v4369	*Cx*. *quinquefasciatus* mosquito	Harris	2003	AY712948
Bird3588	Blue jay	Harris	2004	DQ164206
TX5058	Blue jay	Harris	2005	JF415929
M12214	*Cx*. *quinquefasciatus* mosquito	Harris	2005	JF415915
TX5810	Common grackle	Harris	2006	JF415916
M6019	*Cx*. *quinquefasciatus* mosquito	Harris	2006	JF415930
TX6276	Northern mockingbird	Harris	2006	JF415916
TX6647	Blue jay	Harris	2007	JF415917
TX6747	Blue jay	Harris	2007	JF415918
M19433	*Aedes albopictus* mosquito	Harris	2007	JF415919
TX7191	Blue jay	Harris	2007	JF415920
TX7558	Blue jay	Harris	2008	JF415921
M37012	*Cx*. *quinquefasciatus* mosquito	Harris	2009	JF415922
M37906	*Cx*. *quinquefasciatus* mosquito	Harris	2009	JF415923
TX7827	Blue jay	Harris	2009	JF415924
M38488	*Ae*. *albopictus* mosquito	Harris	2009	JF415925
M20140	*Cx*. *quinquefasciatus* mosquito	Harris	2009	JF415926
M20141	*Ae*. *albopictus* mosquito	Harris	2009	JF415927
M20122	*Cx*. *quinquefasciatus* mosquito	Harris	2009	JF415928

### Harris County Isolates, 2005–2009

#### Nucleotide Changes

The genome of WNV is a single-stranded, positive-sense RNA molecule; the NY99 strain contains 11,029 nt. The genome encodes a 5′ UTR (genomic nt 1–96) and a 3′ UTR (genomic nt 10,396–11,029). The UTRs flank a single ORF that encodes 10 proteins; 3 structural proteins (C, prM/M, and E) and 7 nonstructural proteins (NS1, NS2A, NS2B, NS3, NS4A, NS4B, and NS5).

When compared with NY99, the prototype WNV strain for North America, the 17 WNV isolates we analyzed in this study had 38–60 nt (0.35%–0.54%) differences; most changes were synonymous. Nine of the 13 conserved nt changes characteristic of the NA/WN02 genotype were found in all newly sequenced isolates (Table 2). One 2006 isolate (TX6276), two 2007 isolates (TX6747 and TX7191), and six of seven 2009 isolates (M37012, M37906, M39488, M20140, M20141, and M20122) encoded a C at nt 660 and 6238, which was identical to that in the NY99 strain. Two isolates (M12214 from 2005 and M19433 from 2007) encoded a C at nt 6426, and 2 isolates (TX5810 and M6019, both from 2006) encoded a U at nt 9352, again identical to the NY99 strain. One 2005 isolate (TX5058) contained a 6-nt (nt 10471–10476) deletion in the 3′ UTR, and one 2007 isolate (TX7191) and two 2009 isolates (M37906 and TX7827) contained a 1-nt deletion at nt position 49/50 in the 5′ UTR.

**Table 2:** Nucleotide sequence changes in West Nile virus NA/WN02 genotype, Upper Texas Gulf Coast, USA*

Strain	Year	prM		E			NS2A			NS3					NS4B		NS5			3′ UTR
		660		1442	2466		3774	4146		4803	6138	6238	6426		6996		7938	9352		10851
NY99	1999	C		U	C		U	A		C	C	C	C		C		U	C		A
Bird 114	2002	U		C†	U		U	G		U	U	U	U		U		C	U		G
M12214	2005	U		C	U		U	G		U	U	U	**·**		U		C	U		G
TX5058		U		C	U		U	G		U	U	U	U		U		C	U		G
TX5810	2006	U		C	U		U	G		U	U	U	U		U		C	**·**		G
M6019		U		C	U		U	G		U	U	U	U		U		C	**·**		G
TX 6276		**·**		C	U		U	G		U	U	**·**	U		U		C	U		G
TX 6647	2007	U		C	U		U	G		U	U	U	U		U		C	U		G
TX6747		**·**		C	U		U	G		U	U	**·**	U		U		C	U		G
M19433		U		C	U		U	G		U	U	U	**·**		U		C	U		G
TX7191		**·**		C	U		U	G		U	U	**·**	U		U		C	U		G
TX 7558	2008	U		C	U		U	G		U	U	U	U		U		C	U		G
M 37012	2009	**·**		C	U		U	G		U	U	**·**	U		U		C	U		G
M 37906		**·**		C	U		U	G		U	U	**·**	U		U		C	U		G
TX 7827		U		C	U		U	G		U	U	U	U		U		C	U		G
M 39488		**·**		C	U		U	G		U	U	**·**	U		U		C	U		G
M 20140		**·**		C	U		U	G		U	U	**·**	U		U		C	U		G
M 20141		**·**		C	U		U	G		U	U	**·**	U		U		C	U		G
M20122		**·**		C	U		U	G		U	U	**·**	U		U		C	U		G

*PrM, premembrane; E, envelope; NS, nonstructural; UTR, untranslated region. Values indicate nucleotide position within each gene. Dots indicate no change from NY99 isolate. †Encodes for amino acid substitution E-V159A.

#### Amino Acid Substitutions

Deduced amino acid sequences were compared and substitutions were identified for 41 residues (2 in C, 4 in prM/M, 6 in E, 0 in NS1, 6 in NS2A, 2 in NS2B, 8 in NS3, 2 in NS4A, 4 in NS4B, and 7 in NS5); each isolate contained 3–7 substitutions (Table A1). Eight (C-T109I, E- T70I, E-V159A, E-I460M/L, NS2A-R98G, NS2A-A137V, NS4A-A85T, and NS4B-I240M) of the 41 substitutions were found in >1 isolate. All isolates contained the E-V159A substitution present in the NA/WN02 genotype.

### Upper Texas Gulf Coast Region Isolates, 2002–2009

WNV was first detected in the Upper Texas Gulf Coast region in 2002 (*9*). During 2002–2004, isolates from the Upper Texas Gulf Coast region were divided genetically into 3 groups (groups 1–3) (*12*) and showed 0.30%–0.40% divergence compared with NY99. Isolates from 2005–2009 (groups 4–6; see below for their definitions) have significantly greater divergence (0.40%–0.70%; p<9.4 × 10^–9^) from NY99 (Table 3). When compared with isolates from 2002–2004 (groups 1–3), we found that recent Upper Texas Gulf Coast isolates from 2005–2009 (groups 4–6) have nucleotide divergence rates ranging from 0.50% to 0.80%. Because of the high number of synonymous nucleotide mutations, the deduced amino acid sequences of all isolates exhibited a higher level of conservation; divergence rates ranged from 0.10% to 0.30% compared with NY99.

**Table 3:** Percentage nucleotide and amino acid sequence divergence of West Nile virus isolates, Upper Texas Gulf Coast, USA, 2002–2009*

Group†	NY99	Group 1	Group 2	Group 3	Group 4	Group 5	Group 6
NY99		0.1–0.2	0.1–0.2	0.1	0.1–0.2	0.2	0.2–0.3
Group 1	0.3–0.4		0.1–0.3	0.1–0.3	0.1–0.3	0.2–0.3	0.2–0.4
Group 2	0.3	0.3–0.4		0.1–0.2	0.2–0.3	0.2–0.3	0.2–0.3
Group 3	0.3	0.1–0.4	0.2–0.3		0.1–0.3	0.2–0.3	0.2–0.3
Group 4	0.5–0.6	0.5–0.7	0.5–0.6	0.5–0.6		0.2–0.3	0.2–0.3
Group 5	0.5–0.7	0.5–0.8	0.4–0.7	0.4–0.7	0.7–1.0		0.3
Group 6	0.4–0.5	0.4–0.6	0.4–0.5	0.4–0.5	0.7–0.8	0.6–0.8	

*Amino acid sequence divergence is shown above the diagonal, and nucleotide sequence divergence is shown in below the diagonal**.** †Group 1: bird 114 (2002), bird 1171 (2003), bird 1153 (2003); group 2: bird 1519 (2003), v4369 (2003), v4095 (2003), bird 1881 (2003), v4380 (2003); group 3: bird 1576 (2003), bird 1175 (2003), TX2003; group 4: TX6376 (2006), M20141 (2009), M20140 (2009), M37906 (2009), M39488 (2009), M20122 (2009), M37102 (2009); group 5: M12214 (2005), M19433 (2007), TX6647 (2007), TX7558 (2008); group 6: M6019 (2006), TX5810 (2006).

With the exception of conserved nucleotide mutations in the NA/WN02 genotype, there were a few nucleotide changes or deduced amino acid substitutions that were shared by >1 isolate from 2002–2004 and 1 of the newly sequenced isolates from 2005–2009. Nucleotide changes at 11 positions were shared between >1 isolate from 2002–2004 and the newly sequenced isolates from 2005–2009, with only 1 aa substitution, NS4B-I240M, found in >1 isolate from both groups.

### Phylogenetic Analysis

**Figure 1:**
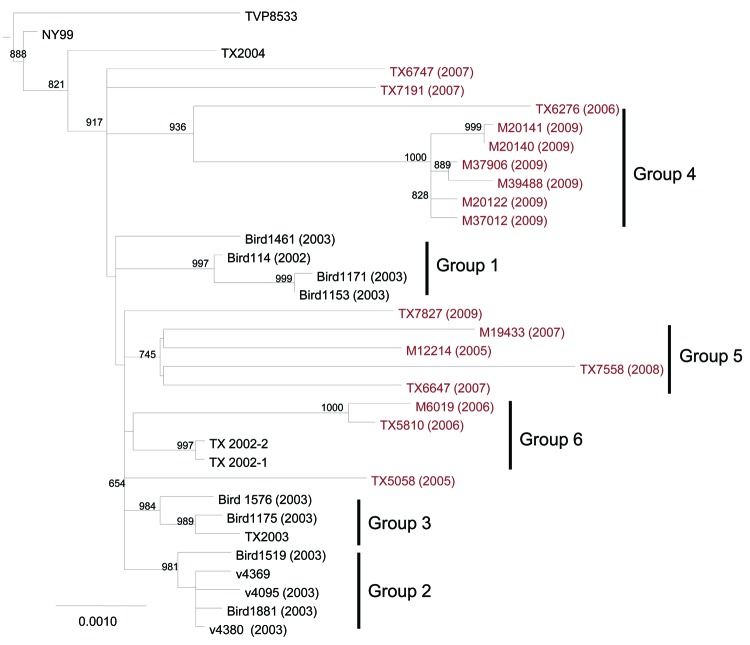
Maximum-likelihood phylogenetic tree of Upper Texas Gulf Coast, USA, West Nile virus isolates, 2002–2009. The tree was inferred from open reading frame sequences of 33 Upper Texas Gulf Coast isolates and NY99 by using PhyML (*17*) and rooted with IS-98 STD. The outgroup has been removed. Bootstrap values are for 1,000 replicates and only values >500 are shown. Groups 1–3 were previously identified by May et al. (*12*). Red, isolates sequenced in this study. Scale bar indicates nucleotide substitutions per site.

Phylogenetic trees were generated by NJ and ML analyses by using only the polyprotein sequence of 34 isolates: the 17 newly sequenced isolates, 16 published sequences of isolates from the Upper Texas Gulf Coast region, and NY99 (Figure 1). Both methods produced trees with similar topology. In addition to groups 1, 2, and 3 identified in Upper Texas Gulf Coast region isolates obtained in 2002–2003 (*12*), we identified 3 other phylogenetic groups in this study.

Group 4 is composed of 6 of 7 isolates from 2009 and 1 isolate from 2006 (TX6276). Group 5 is composed of 4 isolates: 1 from 2005 (M12214), 2 from 2007 (M19433 and TX6647), and 1 from 2008 (TX7558). Group 6 is composed of 2 isolates from 2006 (M6019 and TX5810) and 2 sequenced isolates from 2002. Groups 4 and 5 are supported by high bootstrap values; group 6 has a lower bootstrap value. Within group 4, all 2009 isolates contained an E-I460E substitution. All group 5 isolates contain the amino acid substitution NS4B-A85T, and all isolates in groups 4 and 6 contain the NS4B-I240M substitution. Four isolates did not fall into these 6 groups: TX5058 (2005), TX6747 and TX7191 (2007), and TX7827 (2009).

The TX7828 2009 isolate, which did not fall into group 4 with the other 2009 isolates, is the only 2009 isolate sequenced in this study that was isolated from a bird (blue jay). However, we had only 1 isolate from 2008, and WNV activity was low in Harris County in 2008 (R. Bueno and R. Tesh, unpub. data). This finding may have been caused by Hurricane Ike, which hit the Upper Texas Gulf Coast in September 2008.

**Figure 2:**
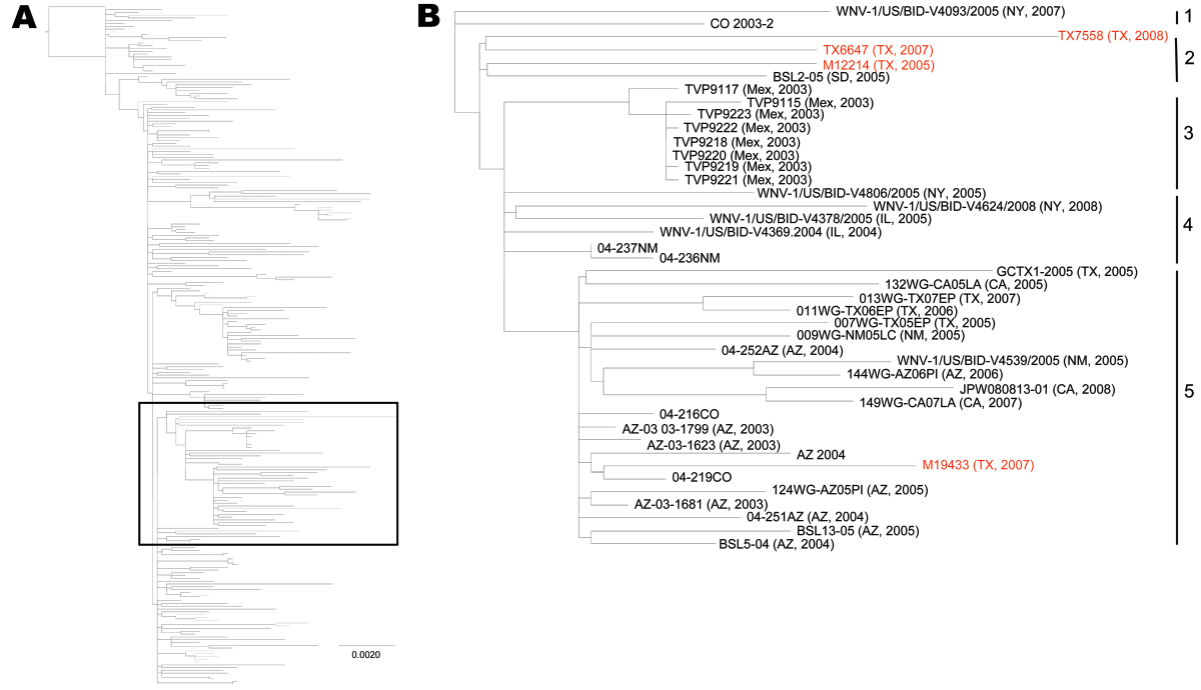
Maximum-likelihood phylogenetic tree showing all published, full open reading frame North American West Nile virus isolates, 2002–2009 (A), and enlargement showing SW/WN03 genotype (B). Red, isolates sequenced in this study. Scale bar in panel A indicates nucleotide substitutions per site. Numbers on the right in panel B indicate groups.

A second phylogenetic analysis was undertaken that used isolates from this study and all published full-length WNV sequences from North America available on GenBank (Figure 2). NJ and ML methods produced trees with similar topology. Within the larger tree, there were analogous groupings of previously and newly sequenced Harris County isolates, as shown in Figure 1. Three of the Harris County groups form distinct clusters of isolates within the NA/WN02 genotype and may represent formation of new genotypes or clusters. Group 1 Harris County isolates (2002–2003) cluster with a grouping of isolates from California from 2003–2008, and group 4 isolates (2006–2009) cluster with several isolates from New York (2008) and 1 isolate from Illinois (2006). Group 5 isolates from Harris County cluster with isolates from the southwestern United States and northern Mexico (called the SW/WN03 genotype because the first isolates were identified in Arizona and Colorado in 2003).

### SW/WN03 Genotype

This genotype is composed of 5 groups on the basis of nucleotide and amino acid sequences and phylogenetic analysis. SW Group 1 is composed of 2 isolates: WNV-1/US/BID-V4093/2007 from New York and CO2003–2 from Colorado. SW Group 2 is composed of 4 isolates, 3 from Texas that were sequenced in this study: M12214 (2005), TX6647 (2007), TX7558 (2008), and BSL2–05 from South Dakota in 2005. SW Group 3 is composed of 6 isolates: 2 from Illinois (WNV-1/US/BID-V4369/2004 and WNV-1/US/BID-V4378/2005), 2 from New York (WNV-1/US/BID-V4806/2005 and WNV-1/US/BID-V4624/2008), and 2 from New Mexico (04–237NM and 04–238NM). SW Group 4 is composed of 8 isolates from Mexico in 2003 (TVP9115, TVP9118–TVP9222). SW Group 5 is the largest and is composed of 22 isolates: 10 from Arizona (2003–2006), 2 from New Mexico (2005), 2 from Colorado (2004), 3 from California (2005, 2007–2008), and 5 from Texas (M19433, which was sequenced in this study, and 4 isolates from west Texas).

Further examination of sequences within the SW/WN03 genotype showed that they share some or all of a signature of 13 nt changes (different from those of the NA/WN02 genotype), including 2 aa substitutions, NS4B-A85T and NS5-K314R (Table A2). Isolates in SW group 1 contain 4 of the 13 changes (nt positions 6238, 6721, 7269, and 9264). SW group 2 isolates contain 5 changes (nt positions 6238, 6721, 8550, 9264, and 9660). SW group 3 isolates have 7 changes (nt positions 1320, 6238, 6721, 8550, 8621, 9264, and 9660). SW group 4 isolates have 6 changes (nt positions 1320, 6238, 6721, 8550, 8621, and 9660). SW group 5 isolates have all 13 nt changes (nt positions 1320, 1974, 3399, 6238, 6721, 6765, 6936, 7269, 8550, 8621, 9264, 9660, and 10062). Isolates in SW groups 1 and 2 contain only 1 (NS4B-A85T) of the 2 aa substitutions, and isolates in SW groups 3, 4, and 5 contain both amino acid substitutions.

### Selection Pressures

Recombination analysis using single-break point analysis was performed on the first 3,333 codons of the ORF of the North American WNV alignment to rule out recombination before performing selection pressure analysis. As expected, no evidence of recombination was detected.

Selection pressures on the WNV genome were examined by using 3 methods: SLAC, FEL, and IFEL (Table 4). These methods estimated the ratio of nonsynonymous (d_N_) to synonymous (d_S_) amino acid substitutions in 10 datasets representing the first 9,999 nt (3,333 aa residues) of the ORF and each protein (C, prM/E, NS1, NS2A, NS2B, NS3, NS4A, NS4B, and NS5) for the complete North American WNV alignment of 244 genomes. On examination of the ORF, 3 residues were identified for positive selection in >2 of the 3 methods. E-V431I (codon position 721 in ORF) was identified by FEL (p = 0.057) and IFEL (p = 0.072), NS2A-A224V/T (codon position 1367 in ORF) was identified by SLAC (p = 0.087) and FEL (p = 0.096), and NS4A-A85T (codon position 2209 in ORF) was identified by SLAC (p = 0.087), FEL (p = 0.011), and IFEL (p = 0.067). When selection analysis was performed on each gene, only E-V431I was identified for positive selection (FEL, p = 0.059 and IFEL, p = 0.065). An additional residue, NS5-K314R, was also identified for positive selection by FEL (p = 0.042) and IFEL (p = 0.042).

**Table 4:** Positive and negative selection results for West Nile virus isolates, Upper Texas Gulf Coast, USA, 2002–2009*

Protein	Amino acid residues relative to ORF	Length of protein, aa	Overall d_N_/d_S_	Single-likelihood ancestor counting†			Fixed effects likelihood†			Internal fixed effects likelihood†	
				Positive selection	Negative selection		Positive selection	Negative selection		Positive selection	Negative selection
ORF	1–3,333‡	3,333	0.110	2	246		8	619		16	25
C	1–123	123	0.270	0	2		0	9		0	2
prM	124–290	166	0.134	0	7		0	25		1	3
E	291–791	500	0.119	0	15		1	72		1	3
NS1	792–1143	351	0.134	0	17		1	48		4	4
NS2A	1144–1374	230	0.130	0	15		1	48		1	3
NS2B	1375–1505	130	0.118	0	6		0	19		0	1
NS3	1506–2124	618	0.083	0	42		0	101		0	6
NS4A	2125–2273	148	0.135	0	7		1	22		0	3
NS4B	2274–2522	248	0.112	0	13		0	49		0	8
NS5	2529–3433	904	0.098	0	61		2	148		4	10

*ORF, open reading frame; d_N_, nonsynonymous nucleotide substitutions; d_S_, synonymous nucleotide substitutions; C, capsid; prM, premembrane; E, envelope; NS, nonstructural. †No. sites where p<0.1. ‡Only the first 3,333 aa residues of the ORF were used for these analyses because of program constraints on alignment size.

NS4A-A85T and NS5-K314R are the 2 aa residues that identify the SW/WN03 genotype described. Residue E-V431I is found in a California cluster (California isolates 2003–2008). Substitutions at NS2A-224 (codon position 1367 in ORF) are found in 5 NY99 genotype isolates (A224T) and 4 SW/NA03 genotype isolates (A224V).

## Discussion

To date, most genetic and phylogenetic studies of WNV have focused on partial genome sequencing, primarily of the E protein gene. Although studies of the E protein gene are helpful in understanding the evolution of WNV in North America, they provide few phylogenetically informative sites; analysis of genomic sequences is more informative (*5**,**26**–**32*). Similarly, although many studies have examined the evolutionary dynamics of WNV soon after its introduction into North America, only 1 published study has examined isolates since 2006 (*33*). To our knowledge, none have been published that examined isolates from 2007 or more recently. For these reasons, we examined evolution of WNV by using genomic sequences from 1999–2009. We focused on the Upper Texas Gulf Coast region because of availability of multiple isolates from the same localities each year since the first detection of the virus in Texas in 2002. These isolates were obtained as part of an ongoing surveillance program of WNV activity in Harris County.

The isolates sequenced in this study demonstrate that the NA/WN02 genotype has been maintained during 2002–2009 in Harris County. All 17 isolates sequenced contained 9 of 13 nt changes associated with the NA/WN02 genotype reported by Davis et al. (*5*), including the amino acid substitution E-V159A. However, since 2005, reversion to the NY99 genotype was seen at 4 nt positions. Nine isolates contained a C at nt positions 660 and 6238, three isolates had a U at nt position 6426, and two isolates had a C at nt position 9352.

Although isolates sequenced in our study display a high degree of similarity, they have major differences. It appears that >3 genetic groups of isolates were co-circulating in Harris County over the study period. Thus, there is continued genetic diversity of WNV over time, at least in the Upper Texas Gulf Coast region, rather than the genetic homeostasis in North America, which was proposed on the basis of using E gene sequences of viruses isolated through 2005 (*13*). One group, group 4, contains isolates from 2006 and 2009. A second group, group 5, contains isolates from 2005, 2007, and 2008. A third group, group 6, contains isolates from 2006 plus 2 sequenced isolates from 2002. All other 2002–2005 isolates sequenced previously fall into other groups (groups 1–3) (*12*). Four isolates, TX5058 from 2005, TX6747 and TX7191 from 2007, and TX7827 from 2009, did not fall into any of the 6 groups and may represent single isolates that did not have any advantage and thus became extinct.

When compared with all North American WNV isolates, we found 3 distinctive clusters of isolates within the NA/WN02 genotype. Each cluster contained several isolates from the Upper Texas Gulf Coast region, in addition to other isolates. The first cluster contains group 4 isolates, in addition to four 2008 isolates from New York and one 2006 isolate from Illinois. The second cluster is composed primarily of isolates from California, in addition to group 1 Upper Texas Gulf Coast isolates and 3 additional isolates from Colorado, Connecticut, and Illinois. Group 5 isolates from Harris County cluster with isolates from the southwestern United States (Arizona and Colorado) and from northern Mexico, which were first identified in 2003. This SW/WN03 genotype shares some or all of 13 nt changes, which encode for 2 aa substitutions.

Our data indicate that this genotype is spreading into new areas. It has been identified in California, Illinois, New Mexico, New York, North Dakota, and Texas since 2003. Two of these clusters, the California cluster and the cluster we have called the SW/WN03 genotype, are further supported by using selection analysis. This analysis has shown that there is potential for positive selection at E-V431I in the California cluster and at both of the amino acid substitutions (NS4A-A85T, NS5-K314R) in the SW/WN03 genotype. This finding further provides evidence of the potential role of this emerging genotype.

Potential roles of single amino acid substitutions within the WNV genome should also be noted. The single amino acid change, E-V159A, which occurred in the NA/WN02 genotype, was shown to decrease the extrinsic incubation period of the virus in mosquitoes, which enabled that genotype to displace the NY99 genotype (*6*). Brault et al. (*34*) reported that the NS3-T249P substitution increased virulence in American crows. The NS3-T249P substitution has undergone positive selection but the E-V159A change has not, yet both cause phenotypic changes. We speculate that positive selection of NS4A-A85T and NS5-K314R induces a phenotypic change in WNV.

Previous studies in our laboratory that focused on the E protein gene concluded that WNV is experiencing a genetic stasis or decrease in its growth rate after establishment of the NA/WN02 genotype (*13*). However, none of these studies have phylogenetically examined the entire genome of WNV. This study of genomic sequences demonstrates evolution of WNV, at least in the Upper Texas Gulf Coast region, and potential emergence of a new genotype in the southwestern United States (SW/WN03 genotype). Further experiments are needed to investigate potential phenotypic changes that occur in conjunction with the noted genotype changes and to determine if the SW/WN03 genotype will replace the current dominant NA/WN02 genotype.

## Appendix

**Table A1:** Deduced amino acid substitutions for West Nile virus isolates, Upper Texas Gulf Coast, USA*

Gene	Position	1999		2005			2006				2007					2008		2009						
		NY99		M12214	TX5058		TX5810	M6019	TX6276		TX6647	TX6747	M19433	TX7191		TX7558		M37012	M37906	TX7827	M38488	M20140	M20141	M20122
C	39*	D		**·**	**·**		**·**	N	**·**		**·**	**·**	**·**	**·**		**·**		**·**	**·**	**·**	**·**	**·**	**·**	**·**
	109	T		**·**	**·**		**·**	**·**	**·**		**·**	**·**	**·**	**·**		**·**		**·**	**·**	**·**	**·**	I	I	**·**
PrM/M	88	R		**·**	**·**		**·**	K	**·**		**·**	**·**	**·**	**·**		**·**		**·**	**·**	**·**	**·**	**·**	**·**	**·**
	122	V		**·**	**·**		**·**	**·**	**·**		I	**·**	**·**	**·**		**·**		**·**	**·**	**·**	**·**	**·**	**·**	**·**
	140	V		**·**	**·**		**·**	**·**	**·**		**·**	I	**·**	**·**		**·**		**·**	**·**	**·**	**·**	**·**	**·**	**·**
	156	V		**·**	**·**		**·**	**·**	**·**		**·**	**·**	**·**	**·**		**·**		**·**	**·**	I	**·**	**·**	**·**	**·**
E	49	E		**·**	**·**		**·**	**·**	K		**·**	**·**	**·**	**·**		**·**		**·**	**·**	**·**	**·**	**·**	**·**	**·**
	70	T		**·**	**·**		**·**	**·**	**·**		**·**	**·**	**·**	**·**		**·**		**·**	I	**·**	I	**·**	**·**	**·**
	159	V		A	A		A	A	A		A	A	A	A		A		A	A	A	A	A	A	A
	431	V		**·**	**·**		**·**	**·**	**·**		**·**	**·**	**·**	**·**		**·**		**·**	**·**	**·**	**·**	**·**	**·**	F
	460	I		**·**	**·**		**·**	**·**	M		**·**	**·**	**·**	**·**		**·**		L	L	**·**	L	L	L	L
	475	N		**·**	**·**		**·**	S	**·**		**·**	**·**	**·**	**·**		**·**		**·**	**·**	**·**	**·**	**·**	**·**	**·**
NS2A	90	M		**·**	**·**		**·**	**·**	**·**		V	**·**	**·**	**·**		**·**		**·**	**·**	V	**·**	**·**	**·**	**·**
	98	R		**·**	**·**		G	G	**·**		**·**	**·**	**·**	**·**		**·**		**·**	**·**	**·**	**·**	**·**	**·**	**·**
	118	Y		**·**	**·**		**·**	**·**	**·**		**·**	**·**	**·**	**·**		**·**		**·**	**·**	**·**	**·**	**·**	**·**	H
	124	I		V	**·**		**·**	**·**	**·**		**·**	**·**	**·**	**·**		**·**		**·**	**·**	**·**	**·**	**·**	**·**	**·**
	137	A		**·**	**·**		**·**	**·**	**·**		**·**	**·**	**·**	**·**		**·**		**·**	**·**	**·**	**·**	V	V	**·**
	224	A		V	**·**		**·**	**·**	**·**		**·**	**·**	**·**	**·**		**·**		**·**	**·**	**·**	**·**	**·**	**·**	**·**
NS2B	41	A		**·**	**·**		**·**	**·**	**·**		**·**	**·**	**·**	**·**		V		**·**	**·**	**·**	**·**	**·**	**·**	**·**
	99	M		**·**	**·**		**·**	**·**	**·**		**·**	**·**	**·**	T		**·**		**·**	**·**	**·**	**·**	**·**	**·**	**·**
NS3	106	V		**·**	**·**		**·**	**·**	**·**		**·**	**·**	**·**	**·**		**·**		**·**	**·**	**·**	A	**·**	**·**	**·**
	160	S		**·**	**·**		**·**	**·**	**·**		**·**	**·**	A	**·**		**·**		**·**	**·**	**·**	**·**	**·**	**·**	**·**
	162	I		**·**	**·**		**·**	**·**	**·**		**·**	**·**	**·**	M		**·**		**·**	**·**	**·**	**·**	**·**	**·**	**·**
	213	I		**·**	**·**		**·**	**·**	**·**		**·**	**·**	**·**	**·**		**·**		**·**	**·**	**·**	M	**·**	**·**	**·**
	355	Y		**·**	**·**		**·**	**·**	**·**		**·**	**·**	**·**	**·**		**·**		**·**	**·**	H	**·**	**·**	**·**	**·**
	365	S		G			**·**	**·**	**·**		**·**	**·**	**·**	**·**		**·**		**·**	**·**	**·**	**·**	**·**	**·**	**·**
	486	F		**·**	L		**·**	**·**	**·**		**·**	**·**	**·**	**·**		**·**		**·**	**·**	**·**	**·**	**·**	**·**	**·**
	562	R		**·**	**·**		**·**	**·**	**·**		K	**·**	**·**	**·**		**·**		**·**	**·**	**·**	**·**	**·**	**·**	**·**
NS4A	65	M		**·**	**·**		**·**	**·**	**·**		**·**	T	**·**	**·**		**·**		**·**	**·**	**·**	**·**	**·**	**·**	**·**
	85	A		T	**·**		**·**	**·**	**·**		T	**·**	T	**·**		T		**·**	**·**	**·**	**·**	**·**	**·**	**·**
NS4B	33	L		**·**	**·**		**·**	F	**·**		**·**	**·**	**·**	**·**		**·**		**·**	**·**	**·**	**·**	**·**	**·**	**·**
	116	T		**·**	**·**		**·**	**·**	**·**		**·**	A	**·**	**·**		**·**		**·**	**·**	**·**	**·**	**·**	**·**	**·**
	176	I		**·**	**·**		**·**	**·**	V		**·**	**·**	**·**	**·**		**·**		**·**	**·**	**·**	**·**	**·**	**·**	**·**
	240	I		**·**	**·**		M	M	M		**·**	**·**	**·**	**·**		**·**		M	M	**·**	M	M	M	M
NS5	21	K		**·**	**·**		**·**	**·**	**·**		R	**·**	**·**	**·**		**·**		**·**	**·**	**·**	**·**	**·**	**·**	**·**
	91	M		**·**	**·**		**·**	**·**	**·**		**·**	**·**	V	**·**		**·**		**·**	**·**	**·**	**·**	**·**	**·**	**·**
	314	K		**·**	**·**		**·**	**·**	**·**		**·**	**·**	R	**·**		**·**		**·**	**·**	**·**	**·**	**·**	**·**	**·**
	395	M		**·**	**·**		**·**	**·**	**·**		**·**	**·**	**·**	**·**		**·**		**·**	I	**·**	**·**	**·**	**·**	**·**
	422	R		**·**	K		**·**	**·**	**·**		**·**	**·**	**·**	**·**		**·**		**·**	**·**	**·**	**·**	**·**	**·**	**·**
	640	L		**·**	**·**		P	**·**	**·**		**·**	**·**	**·**	**·**		**·**		**·**	**·**	**·**	**·**	**·**	**·**	**·**
	661	S		**·**	**·**		T	**·**	**·**		**·**	**·**	**·**	**·**		**·**		**·**	**·**	**·**	**·**	**·**	**·**	**·**
	860	A		**·**	**·**		**·**	**·**	**·**		**·**	**·**	**·**	T		**·**		**·**	**·**	**·**	**·**	**·**	**·**	**·**

*C, capsid; PrM/M, premembrane/membrane; E, envelope, NS nonstructural. Dot indicates no change from NY99 isolate.

**Table A2:** Genotype nucleotide changes in SW/WN03 isolate of West Nile virus, Upper Texas Gulf Coast, USA*

Strain	State	Year	Group	E			NS1		NS3		NS4A			NS4B			NS5					3′ UTR
1320	1974	3399	6238	6721†	6765	6936	7269	8550	8621‡	9264	9660	10062
NY99	NY	1999		A	C		U		C		G	**U**		U	U		C	A	U	C		U
CO 2003–2	CO	2003	1	·	·		·		U§		A	·		·	C		·	·	C	·		·
HM488201	NY	2007		·	·		·		U		A	·		·	C		·	·	C	·		·
**M12214**	**TX**	**2005**	**2**	**·**	**·**		**·**		**U**		**A**	**·**		**·**	**·**		**U**	**·**	**C**	**U**		**·**
BSL2–05	SD	2005		·	·		·		U		A	·		·	·		U	·	C	U		·
**TX 6647**	**TX**	**2007**		**·**	**·**		**·**		**U**		**A**	**·**		**·**	**·**		**U**	**·**	**C**	**U**		**·**
**TX 7558**	**TX**	**2008**		**·**	**·**		**·**		**U**		**A**	**·**		**·**	**·**		**U**	**·**	**C**	**U**		**·**
HM488191	IL	2004	3	G	·		·		U		A	·		·	·		U	G	C	U		·
04–237NM	NM	2004		G	·		·		U		A	·		·	·		U	G	C	U		·
04–236NM	NM	2004		G	·		·		U		A	·		·	·		U	G	C	U		·
HM488198	IL	2005		G	·		·		U		A	·		·	·		U	G	C	U		·
HM756675	NY	2005		G	·		·		U		A	·		·	·		U	G	C	U		·
HM488239	NY	2008		G	·		·		U		A	·		·	·		U	G	C	U		·
TVP9117	MEX	2003	4	G	·		·		·		A	·		·	·		U	G	·	U		·
TVP9223	MEX	2003		G	·		·		U		A	·		·	·		U	G	·	U		·
TVP9115	MEX	2003		G	·		·		U		A	·		·	·		U	G	·	U		·
TVP9222	MEX	2003		G	·		·		U		A	·		·	·		U	G	·	U		·
TVP9218	MEX	2003		G	·		·		U		A	·		·	·		U	G	·	U		·
TVP9220	MEX	2003		G	·		·		U		A	·		·	·		U	G	·	U		·
TVP9219	MEX	2003		G	·		·		U		A	·		·	·		U	G	·	U		·
TVP9221	MEX	2003		G	·		·		U		A	·		·	·		U	G	·	U		·
AZ-03–1623	AZ	2003	5	G	U		C		U		A	C		C	C		U	G	C	U		C
AZ-03 03–1799	AZ	2003		G	U		C		U		A	C		C	C		U	G	C	U		C
AZ-03–1681	AZ	2003		G	U		C		U		A	C		C	C		U	G	C	U		C
BSL5–04	AZ	2004		G	U		C		U		A	C		C	C		U	G	C	U		C
04–252AZ	AZ	2004		G	U		C		U		A	C		C	C		U	G	C	U		C
04–251AZ	AZ	2004		G	U		C		U		A	C		C	C		U	G	C	U		C
AZ2004	AZ	2004		G	U		C		U		A	C		C	C		U	G	C	U		C
04–216CO	CO	2004		G	U		C		U		A	C		C	C		U	G	C	U		C
04–219CO	CO	2004		G	U		C		U		A	C		C	C		U	G	C	U		C
BSL13–05	AZ	2005		G	U		C		U		A	C		C	C		U	G	C	U		C
142WG-AZ05PI	AZ	2005		G	U		C		U		A	C		C	C		U	G	C	U		C
132WG-CA05LA	CA	2005		G	U		C		U		A	C		C	C		U	G	C	·		C
HM756677	NM	2005		G	U		C		U		A	C		C	C		U	G	C	U		C
009WG-NM05LC	NM	2005		G	U		C		U		A	C		C	C		U	G	C	U		C
GCTX1–2005	TX	2005		G	U		C		U		A	C		C	C		U	G	·	·		C
007WG-TX05EP	TX	2005		G	U		C		U		A	C		C	C		U	G	C	U		C
144WG-AZ06PI	AZ	2006		G	U		C		U		A	C		·	C		U	G	C	U		C
011WG-TX06EP	TX	2006		G	U		C		U		A	C		C	C		U	G	C	U		C
194WG-CA07LA	CA	2007		G	U		C		U		A	C		C	C		U	G	C	U		C
013WG-TX07EP	TX	2007		G	U		C		U		A	C		C	C		U	G	C	U		C
**M19433**	**TX**	**2007**		**G**	**U**		**C**		**U**		**A**	**C**		**C**	**C**		**U**	**G**	**C**	**U**		**C**
JPW080814–01	CA	2008		G	U		C		U		A	C		C	C		U	G	C	U		C

*E, envelope; NS, nonstructural; UTR, untranslated region; NY, New York; CO, Colorado; TX, Texas; SD, South Dakota; IL, Illinois, NM, New Mexico; MEX, Mexico, AZ, Arizona; CA, California. Dots indicate no change from NY99 isolate. Isolates in **boldface** were sequenced in this study. †NS4A-A85T. ‡NS5-K314R. §Nucleotide changes compared with NY99 isolate.
